# Analysis of Racial and Ethnic Diversity of Population Served and Imaging Used in US Children’s Hospital Emergency Departments

**DOI:** 10.1001/jamanetworkopen.2022.13951

**Published:** 2022-06-02

**Authors:** Margaret E. Samuels-Kalow, Heidi G. De Souza, Mark I. Neuman, Elizabeth Alpern, Jennifer R. Marin, Jennifer Hoffmann, Matt Hall, Paul L. Aronson, Alon Peltz, Jordee Wells, Colleen K. Gutman, Harold K. Simon, Kristen Shanahan, Monika K. Goyal

**Affiliations:** 1 Department of Emergency Medicine, Massachusetts General Hospital, Harvard Medical School, Boston; 2 Children’s Hospital Association, Lenexa, Kansas; 3 Division of Emergency Medicine, Boston Children’s Hospital, Boston, Massachusetts; 4 Division of Emergency Medicine, Department of Pediatrics, Ann & Robert H. Lurie Children’s Hospital of Chicago, Chicago, Illinois; 5 Department of Pediatrics, University of Pittsburgh School of Medicine, Pittsburgh, Pennsylvania; 6 Department of Emergency Medicine, University of Pittsburgh School of Medicine, Pittsburgh, Pennsylvania; 7 Department of Radiology, University of Pittsburgh School of Medicine, Pittsburgh, Pennsylvania; 8 Department of Pediatrics, Yale School of Medicine, New Haven, Connecticut; 9 Department of Emergency Medicine, Yale School of Medicine, New Haven, Connecticut; 10 Department of Population Medicine, Harvard Pilgrim Health Care, Harvard Medical School, Boston, Massachusetts; 11 Department of Pediatrics, Boston Children’s Hospital, Boston, Massachusetts; 12 Division of Emergency Medicine, Nationwide Children’s Hospital, Columbus, Ohio; 13 Department of Emergency Medicine, University of Florida College of Medicine, Gainesville; 14 Department of Pediatrics, Emory University School of Medicine, Children’s Healthcare of Atlanta, Atlanta, Georgia; 15 Department of Emergency Medicine, Emory University School of Medicine, Children’s Healthcare of Atlanta, Atlanta, Georgia; 16 Department of Pediatrics, Children’s National Hospital, George Washington University, Washington, DC

## Abstract

**Importance:**

Lower rates of diagnostic imaging have been observed among Black children compared with White children in pediatric emergency departments. Although the racial composition of the pediatric population served by each hospital differs, it is unclear whether this is associated with overall imaging rates at the hospital level, and in particular how it may be associated with the difference in imaging rates between Black and White children at a given hospital.

**Objective:**

To examine the association between the diversity of the pediatric population seen at each pediatric ED and variation in diagnostic imaging.

**Design, Setting, and Participants:**

Cross-sectional analysis of ED visits by patients younger than 18 years at 38 children’s hospitals from January 1, 2016, through December 31, 2019, using data from the Pediatric Health Information System. Data were analyzed from April to September 2021.

**Exposures:**

Proportion of patients from minoritized groups cared for at each hospital.

**Main Outcomes and Measures:**

The primary outcome was receipt of an imaging test defined as radiography, ultrasonography, computed tomography, or magnetic resonance imaging; adjusted odds ratios (aORs) were calculated to measure differences in imaging by race and ethnicity by hospital, and the correlation between the proportion of patients from minoritized groups cared for at each hospital and the aOR for receipt of diagnostic imaging by race and ethnicity was examined.

**Results:**

There were 12 310 344 ED visits (3 477 674 [28.3%] among Hispanic patients; 3 212 915 [26.1%] among non-Hispanic Black patients; 4 415 747 [35.9%] among non-Hispanic White patients; 6 487 660 [52.7%] among female patients) by 5 883 664 pediatric patients (mean [SD] age, 5.84 [5.23] years) to the 38 hospitals during the study period, of which 3 527 866 visits (28.7%) involved at least 1 diagnostic imaging test. Diagnostic imaging was performed in 1 508 382 visits (34.2%) for non-Hispanic White children, 790 961 (24.6%) for non-Hispanic Black children, and 907 222 (26.1%) for Hispanic children (*P* < .001). Non-Hispanic Black patients were consistently less likely to receive diagnostic imaging than non-Hispanic White patients at each hospital, and for all imaging modalities. There was a significant correlation between the proportion of patients from minoritized groups cared for at the hospital and greater imaging difference between non-Hispanic White and non-Hispanic Black patients (correlation coefficient, −0.37; 95% CI, −0.62 to −0.07; *P* = .02).

**Conclusions and Relevance:**

In this cross-sectional study, hospitals with a higher percentage of pediatric patients from minoritized groups had larger differences in imaging between non-Hispanic Black and non-Hispanic White patients, with non-Hispanic White patients consistently more likely to receive diagnostic imaging. These findings emphasize the urgent need for interventions at the hospital level to improve equity in imaging in pediatric emergency medicine.

## Key Points

**Question:**

Is there a correlation between the diversity of pediatric patients served at pediatric EDs and variation in diagnostic imaging by race?

**Findings:**

In this cross-sectional study of 38 children’s hospitals encompassing more than 12 million ED visits, hospitals with a higher percentage of patients from minoritized groups had larger differences in imaging between non-Hispanic Black and non-Hispanic White patients, with non-Hispanic White patients consistently more likely to receive diagnostic imaging.

**Meaning:**

These findings suggest the need for interventions at the hospital level to improve equity in imaging in pediatric emergency medicine.

## Teaser

This cross-sectional study evaluates the association between racial and ethnic diversity of the population served and the likelihood of imaging in US children’s hospital emergency departments.

## Introduction

Multiple studies have demonstrated differences in the utilization of diagnostic imaging in the pediatric emergency department (ED) by patient race. Black pediatric patients have lower rates of imaging compared with White patients for head and abdominal computed tomography (CT) in trauma,^1,2,3^ ultrasonography (US) or CT for abdominal pain,^4,5^ and chest radiograph for bronchiolitis^6^ and asthma.^7^ Lower rates of imaging have also been shown for Hispanic patients^8,9^ compared with non-Hispanic patients. In a national sample of pediatric ED visits, non-Hispanic Black and Hispanic children were less likely to receive diagnostic imaging during pediatric ED visits compared with non-Hispanic White children even after adjustment for relevant confounders.^9^ Similarly, in general EDs, non-Hispanic Black patients have a decreased odds of imaging compared with non-Hispanic White patients.^10^ The higher rate of imaging in non-Hispanic White children does not necessarily indicate higher quality of care; however, the persistent inequities in management highlight the presence of structural and individual biases promoting differential care.

These data add to the growing literature demonstrating variability in health care provision, resource utilization and quality of care associated with patient race and ethnicity for both adults and children. However, the underlying explanation for these differences remains undefined. Understanding the factors underlying these differences is critically important to the design of interventions to reduce disparities in imaging. Several studies have suggested an important role for hospital-level factors as a driver of disparities in care.^11^ One study in adults found that Black patients were more likely to live closer to high-quality hospitals, but they continue to receive surgery at low quality hospitals.^12^ Similarly, Black and Hispanic women are more likely to deliver in hospitals with high complication rates.^13^ Another study reported both between- and within-hospital variation in quality of care in neonatal intensive care units by race and ethnicity.^14^ It remains unknown to what degree similar variation exists because of differences in where children receive emergency care (eg, non-Hispanic Black patients receiving care at facilities that usee imaging at lower rates) or because of children receiving different care at the same hospital (eg, Black patients are less likely to be imaged than non-Hispanic White patients within the same hospital). Improving our understanding of this imaging disparity is critical for prioritizing and guiding interventions to reduce disparities in care. Therefore, the goal of this investigation was to measure potential hospital-level factors associated with differences in diagnostic imaging in the pediatric ED by race and ethnicity.

## Methods

### Data Sources

We conducted a planned secondary analysis of a data set created to examine diagnostic imaging utilization in pediatric EDs.^9^ In brief, we used the Pediatric Health Information System (PHIS; Children's Hospital Association [CHA]), which contains administrative data from 49 tertiary care children’s hospitals in the US. Participating hospitals are in 27 states as well as Washington, DC After excluding 11 hospitals that did not contribute ED data during the entire study period or did not contribute consistent race and ethnicity data, there were 38 EDs in the cohort (eFigure in the Supplement) We included all ED visits from January 1, 2016, through December 31, 2019, by patients younger than 18 years of age. This period was selected to enable use of the *International Statistical Classification of Diseases, Tenth Revision, Clinical Modification (ICD-10-CM)*, which was adopted in 2015 across participating sites. The University of Pittsburgh institutional review board determined that the study did not involve human participants and therefore was exempt from review and informed consent. The study followed the Strengthening the Reporting of Observational Studies in Epidemiology (STROBE) reporting guideline. Data were analyzed from April to September 2021.

### Exposures and Outcome Measures

The primary analysis was conducted at the hospital level. We assessed the racial and ethnic composition of patients served at a given hospital as the primary exposure of interest. In PHIS, race and ethnicity are submitted by hospitals as separate variables for each visit on health care claims according to hospital-specific collection practices. We categorized race and ethnicity into 4 groups, Hispanic of any race, non-Hispanic White, non-Hispanic Black, and non-Hispanic other.^9^ Race was missing in 1.3% of encounters (range across hospitals: 0.0%-8.6%) and classified as non-Hispanic other for analyses. There was substantial heterogeneity in the non-Hispanic other group (162 218 [1.32%] were multiracial; 333 582 [2.71%] were Asian; 30 888 [0.25%] were American Indian; 28 919 ([0.23%] were Native [eg, Alaskan Native]; 648 401[5.27%] were other). Because of this heterogeneity, our primary comparisons were between (1) non-Hispanic Black and non-Hispanic White, (2) Hispanic and non-Hispanic Black, and (3) Hispanic and non-Hispanic White.

For calculation of hospital demographic composition, we used the percentage of all children not classified as non-Hispanic White seen in the ED during the study period. Additional exposures of interest at the hospital level included the percentage of ED visits with Medicaid, overall mean imaging rates, overall annual ED census, and magnetic resonance imaging (MRI) availability (as a proxy for imaging resources) calculated as the number of MRI machines per 10 000 patients. Data on MRI machine access is collected annually by CHA as part of their PROSPECT database.

The primary outcome was the degree of racial and ethnic differences in the receipt of diagnostic imaging at a given hospital. Diagnostic imaging was defined as radiograph, US, CT, or MRI and determined from billing data. For patients who are admitted through the ED, PHIS does not distinguish between imaging performed in the ED and imaging performed as an inpatient on the same day. To be consistent with prior work,^9,15,16^ we defined imaging for admitted patients as follows: if ED arrival time was before 6 pm, we attributed imaging to the ED if it occurred on the day of arrival; if ED arrival time was after 6 pm, we attributed imaging to the ED if it occurred on the day of arrival or the next day.

### Statistical Analysis

To determine the degree of racial and ethnic differences in imaging at a given hospital, we used logistic regression models for each hospital to calculate adjusted odds ratios (aORs) for receipt of any diagnostic imaging as well as individual imaging tests (ie, radiograph, US, CT, MRI) across the 3 racial and ethnic groups^9,17,18^ (non-Hispanic Black and non-Hispanic White, Hispanic and non-Hispanic Black, and Hispanic and non-Hispanic White) at each hospital. As our prior work^9^ has demonstrated persistent associations between race and ethnicity and imaging after adjustment for age, sex, weekend presentation, hour of presentation, insurance type, hospital admission, intensive care unit admission, complex chronic conditions, All Patient Refined–Diagnosis Related Group (3M Healthcare) category, year, distance from hospital, and 3-day revisit, we determined a priori to include those factors in the model. These factors were measured at the patient level.

We subsequently calculated Pearson correlation coefficient between the hospital characteristics described above (percetage of population non-Hispanic White, percentage of population receiving Medicaid, mean imaging rates, yearly ED census, MRI access) and the aOR to examine the association between hospital-level factors and the degree of differences in imaging. All hypothesis testing was 2-sided, with statistical significance defined as *P* < .05. We used SAS, version 9.4 (SAS Institute) for all analyses.

To further examine the role of hospital characteristics in imaging disparities, we constructed a generalized linear mixed effects model treating patient level variables as random effects and hospital level variables as fixed effects with a random intercept for each hospital. For this model, we included only the first visit by a patient to avoid concerns about bias from return visits. For this sensitivity analysis, we focused on the primary result of increased Black-White disparities in imaging at hospitals with higher percentages of patients from minoritized groups and included both hospital mean imaging rates and hospital demographics (percentage of patients from minoritized groups seen in the ED) in the model. We chose mean imaging rate to determine if lower rates of imaging in Black patients were due to differential presentation to hospitals with a lower tendency to image.

## Results

### Cohort Characteristics

There were 12 310 344 ED visits by 5 883 664 patients during the study period. Of the ED visits, 4 415 747 (35.9%) were by non-Hispanic White patients, 3 212 915 (26.1%) were by non-Hispanic Black patients and 3 477 674 (28.3%) were by Hispanic patients. Across 38 hospitals, patients from minoritized groups accounted for 31% to 96% (median [IQR] 66.5% [49.6%-66.5%]) of children in this cohort. The mean [SD] age in years was 5.84 [5.23]; 6 487 660 (52.7%) visits were by male patients and 7 828 197 (65.1%) were by individuals with public insurance (Table 1).

**Table 1.** Characteristics of Cohort by Race and Ethnicity

Characteristic	Encounters, No. (%)				
	All (N = 12 310 344)	Non-Hispanic White (n = 4 415 747)	Non-Hispanic Black (n = 3 212 915)	Hispanic (n = 3 477 674)	Other (n = 1 204 008)
Age, y					
<1	1 963 820 (16.0)	670 167 (15.2)	512 467 (16.0)	565 719 (16.3)	215 467 (17.9)
1-4	4 277 162 (34.7)	1 458 316 (33.0)	1 122 663 (34.9)	1 229 486 (35.4)	466 697 (38.8)
5-12	4 127 237 (33.5)	1 487 040 (33.7)	1 064 314 (33.1)	1 198 014 (34.4)	377 869 (31.4)
13-18	1 942 125 (15.8)	800 224 (18.1)	513 471 (16.0)	484 455 (13.9)	143 975 (12.0)
Sex					
Female	5 822 684 (47.3)	2 093 236 (47.4)	1 538 839 (47.9)	1 637 303 (47.1)	553 306 (46.0)
Male	6 487 660 (52.7)	2 322 511 (52.6)	1 674 076 (52.1)	1 840 371 (52.9)	650 702 (54)
Insurance					
Public	7 828 197 (65.1)	1 862 473 (42.8)	2 520 855 (79.1)	2 685 508 (81.5)	759 361 (64.1)
Private	3 515 241 (29.2)	2 277 774 (52.3)	470 986 (14.8)	407 749 (12.4)	358 732 (30.3)
Other	674 704 (5.6)	210 940 (4.8)	194 402 (6.1)	202 493 (6.1)	66 869 (5.6)
Weekend (vs weekday)	3 553 653 (28.9)	1 331 570 (30.2)	869 358 (27.1)	990 197 (28.5)	362 528 (30.1)
ED arrival time					
8:00 am to 3:59 pm	4 591 397 (37.3)	1 637 360 (37.1)	1 277 172 (39.8)	1 237 508 (35.6)	439 357 (36.5)
4:00 pm to 11:59 pm	6 061 798 (49.3)	2 257 862 (51.1)	1 487 388 (46.3)	1 716 123 (49.4)	600 425 (49.9)
12:00 am to 7:59 am	1 654 830 (13.4)	519 372 (11.8)	447 862 (13.9)	523 682 (15.1)	163 914 (13.6)
Distance from hospital, miles					
>20	3 026 370 (24.7)	649 092 (14.7)	1 212 828 (37.8)	850 502 (24.6)	313 948 (26.3)
10-19	3 492 081 (28.5)	948 937 (21.5)	1 110 973 (34.6)	1 039 141 (30.0)	393 030 (32.9)
5-9	3 051 385 (24.9)	1 285 324 (29.2)	577 339 (18.0)	893 172 (25.8)	295 550 (24.7)
<5	2 698 795 (22.0)	1 520 583 (34.5)	307 738 (9.6)	678 282 (19.6)	192 192 (16.1)
Year^a^					
2016	3 039 997 (24.7)	1 078 919 (24.4)	809 533 (25.2)	867 017 (24.9)	284 528 (23.6)
2017	3 091 895 (25.1)	1 108 991 (25.1)	803 490 (25.0)	876 114 (25.2)	303 300 (25.2)
2018	3 055 126 (24.8)	1 098 989 (24.9)	796 746 (24.8)	858 058 (24.7)	301 333 (25.0)
2019	3 123 326 (25.4)	1 128 848 (25.6)	803 146 (25.0)	876 485 (25.2)	314 847 (26.1)
Admit	1 384 449 (11.2)	645 886 (14.6)	308 394 (9.6)	293 005 (8.4)	137 164 (11.4)
ICU utilization	147 928 (1.2)	65 210 (1.5)	36 314 (1.1)	30 354 (0.9)	16 050 (1.3)
CCC	837 761 (6.8)	350 639 (7.9)	211 072 (6.6)	195 531 (5.6)	80 519 (6.7)
3-d return visit	138 128 (1.1)	54 855 (1.2)	31 333 (1.0)	37 840 (1.1)	14 100 (1.2)
Any imaging	3 527 866 (28.7)	1 508 382 (34.2)	790 961 (24.6)	907 222 (26.1)	321 301 (26.7)
CXR	2 821 020 (22.9)	1 178 071 (26.7)	670 782 (20.9)	714 688 (20.6)	257 479 (21.4)
CT	380 164 (3.1)	185 451 (4.2)	79 555 (2.5)	84 504 (2.4)	30 654 (2.5)
US	689 561 (5.6)	310 866 (7.0)	111 207 (3.5)	201 780 (5.8)	65 708 (5.5)
MRI	85 511 (0.7)	45 479 (1.0)	14 656 (0.5)	17 826 (0.5)	7550 (0.6)

Abbreviations: CCC, complex chronic condition; CT, computed tomography; CXR, chest radiograph; ED, emergency department; ICU, intensive care unit; MRI, magnetic resonance imaging; US, ultrasonography.

^a^ All comparisons of characteristics by race and ethnicity were significant at *P* < .001 except year (*P* = .62).

### Imaging Utilization

**Figure 1.**
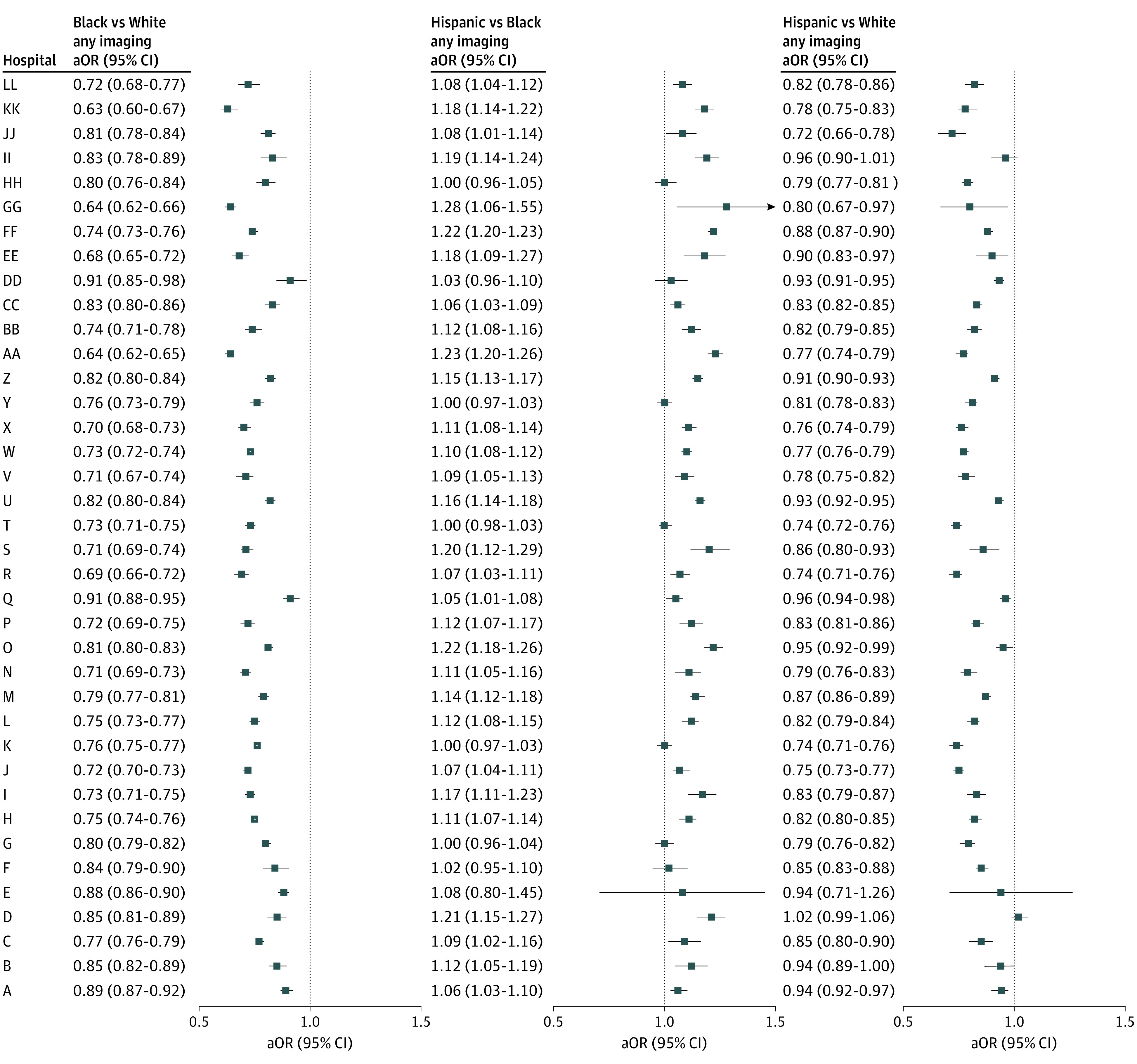
Adjusted Odds Ratios (aOR) of Any Imaging by Hospital

Overall, 3 527 866 (28.7%) visits involved at least 1 diagnostic imaging test; 2 821 020 (22.9%) included radiograph, 380 164 (3.1%) included CT, 689 561 (5.6%) included ultrasonography, and 85 511 (0.7%) included MRI. Diagnostic imaging was performed in 1 508 382 visits (34.2%) for non-Hispanic White children, 790 961 (24.6%) for non-Hispanic Black children, and 907 222 (26.1%) for Hispanic children (*P* < .001). In the unadjusted analyses by hospital, non-Hispanic Black and Hispanic children were less likely to undergo diagnostic imaging than non-Hispanic White children in all but 1 hospital (where the difference was nonsignificant between Hispanic and non-Hispanic White) (eTable 1 in the Supplement). Figure 1 shows the adjusted odds ratios of any imaging, radiograph, US, CT, and MRI. Consistently, non-Hispanic Black patients were less likely to undergo imaging than non-Hispanic White patients across all hospitals and imaging modalities; similarly Hispanic patients were less likely to undergo imaging than non-Hispanic White patients, although the results were less consistent for US and MRI. Additionally, non-Hispanic Black patients were less likely to be imaged than Hispanic patients for all imaging and particularly for ultrasonography (eTable 2 in the Supplement).

### Imaging Utilization and Hospital Characteristics

**Figure 2.**
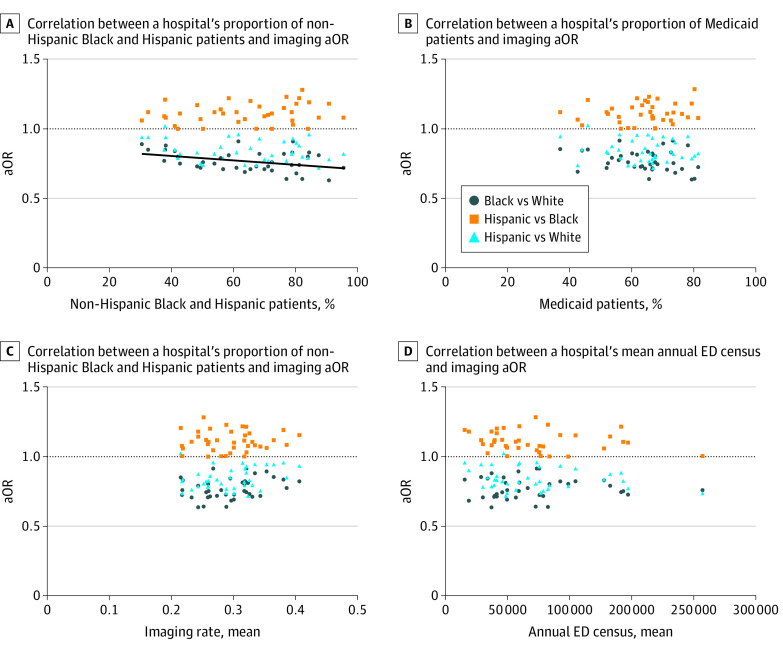
Correlations Between Hospital Characteristics and Adjusted Odds Ratio (aOR) of Any Imaging by Race and Ethnicity aOR for any imaging as compared between race and ethnicity groups (y-axis) and the percentage of patients from minoritized groups cared for at a given hospital (x-axis in panels A and B), where each dot represents an individual hospital. Trend lines are shown only for significant correlations. ED indicates emergency department.

Next, we examined the correlation between hospital characteristics and the aOR for imaging between race and ethnicity groups as a measure of the degree of differences in imaging by the racial and ethnic composition of the population served by hospital. The percentage of the hospital population served that were from minoritized groups was significantly correlated with the aOR for any imaging between non-Hispanic Black and non-Hispanic White children (correlation coefficient, −0.37; 95% CI, −0.62 to −0.07; *P* = .02) (Table 2). In other words, hospitals with a higher percentage of patients from minoritized groups had larger differences in imaging between non-Hispanic Black and non-Hispanic White patients (Figure 2), with non-Hispanic White patients consistently more likely to receive diagnostic imaging. Hospital racial composition was not correlated with the degree of imaging differences between Hispanic children and other racial and ethnic groups, when examining overall imaging rates (Table 2). However, for MRI imaging, hospitals with a larger percentage of patients from minoritized groups had greater odds of imaging non-Hispanic White patients compared with Black or Hispanic patients (Table 2).

**Table 2.** Correlations Between Hospital-Level Factors and aOR of Imaging by Race and Ethnicity

Odds of receiving imaging	Hospital-level factors									
	% Patients from minoritized groups		% Medicaid		Mean imaging rates		Overall yearly ED Census		No. MRI/10 000 patients^a^	
	Correlation coefficient	*P* value	Correlation coefficient	*P* value	Correlation coefficient	*P* value	Correlation coefficient	*P* value	Correlation coefficient	*P* value
Any imaging										
Non-Hispanic Black vs non-Hispanic White^b^	–0.37^c^	.02	–0.26	.11	0.30	.06	0.02	.88	NA	NA
Hispanic vs non-Hispanic Black	0.20	.22	0.15	.37	0.04	.81	–0.18	.28	NA	NA
Hispanic vs non-Hispanic White	–0.28	.09	–0.15	.38	0.28	.09	–0.22	.18	NA	NA
Radiograph imaging										
Non-Hispanic Black vs non-Hispanic White	–0.18	.28	–0.16	.34	0.41	.01	–0.18	.27	NA	NA
Hispanic vs non-Hispanic Black	–0.02	.92	0.12	.48	–0.03	.84	0.00	.99	NA	NA
Hispanic vs non-Hispanic White	–0.28	.09	–0.07	.66	0.33	.05	–0.30	.07	NA	NA
CT imaging										
Non-Hispanic Black vs non-Hispanic White	–0.26	.11	–0.10	.55	0.11	.49	0.07	.65	NA	NA
Hispanic vs non-Hispanic Black	0.10	.55	0.06	.71	–0.16	.35	–0.10	.54	NA	NA
Hispanic vs non-Hispanic White	–0.08	.63	0.04	.81	–0.05	.77	–0.12	.47	NA	NA
US imaging										
Non-Hispanic Black vs non-Hispanic White	–0.08	.62	–0.30	.07	0.07	.69	0.12	.49	NA	NA
Hispanic vs non-Hispanic Black	0.18	.27	0.42	.01	0.06	.71	–0.19	.26	NA	NA
Hispanic vs non-Hispanic White	0.05	.76	0.05	.79	0.05	.75	–0.13	.42	NA	NA
MRI imaging										
Non-Hispanic Black vs non-Hispanic White	–0.44	.01	–0.25	.13	0.10	.54	0.37	.02	–0.21	.456
Hispanic vs non-Hispanic Black	–0.21	.20	0.04	.80	0.21	.20	–0.30	.07	–0.05	.847
Hispanic vs non-Hispanic White	–0.43	.01	–0.05	.77	0.22	.19	–0.10	.56	–0.20	.478

Abbreviations: aOR, adjusted odds ratio; CT, computed tomography; ED, emergency department; MRI, magnetic resonance imaging; NA, not applicable; US, ultrasonography.

^a^ Only includes 47% of the hospitals in the cohort for which the number of MRIs was available.

^b^ The adjusted odds ratios (aOR) for which the correlations are calculated represent the results of generalized linear modeling adjusted for age, sex, weekend presentation, hour of presentation, insurance, hospital admission, intensive care unit admission, hospital site, complex chronic conditions, all patient refined–diagnosis related group category, year, distance from hospital, and 3-day revisit, comparing the receipt of imaging for the first group shown as compared with the second group (eg, Black vs White would be Black patients as the comparison group as compared with White patients as the referent group).

^c^ The table describes the correlation between hospital characteristics (row 1) and the adjusted odds ratios for imaging by race and ethnicity (a measure of the difference in imaging by race, adjusted for potential confounders). Negative correlations mean the comparison group is less likely to be imaged than the referent group as the percentage of the hospital characteristic increases (eg, a negative correlation indicates that, in hospitals with higher percentages of patients from minoritized groups, non-Hispanic Black patients are less likely to be imaged, resulting in larger Black-White differences in imaging rates).

There was no significant correlation between the other hospital-level characteristics and the aOR of imaging by race and ethnicity when examining overall imaging rates. We did, however, find correlations for some specific imaging studies. We found that an increased percentage of patients receiving Medicaid was associated with a greater difference in US imaging between Hispanic and non-Hispanic Black children (correlation coefficient, 0.42; 95% CI, 0.11 - 0.65; *P* = .009). A higher hospital mean imaging rate was correlated with a greater difference in the likelihood of radiograph imaging in non-Hispanic Black compared with non-Hispanic White children (correlation coefficient, 0.41; 95% CI, 0.10 - 0.64; *P* = .01). Furthermore, annual ED census was correlated with a greater difference in the likelihood of MRI in non-Hispanic Black children compared with non-Hispanic White children (correlation coefficient, 0.37; 95% CI, 0.04 - 0.62; *P* = .03), and no significant associations were seen based on MRI availability.

### Sensitivity Analysis

When examining imaging obtained at the first visit across the cohort, non-Hispanic Black children were significantly less likely to receive imaging than non-Hispanic White children (aOR, 0.77; 95% CI, 0.74-0.79). This remained significant even after adjustment for a priori specified confounders including hospital propensity to image (Table 3).

**Table 3.** Mixed Effects Model for the Association of Race and Ethnicity and Receipt of Any Imaging

Characteristic	aOR (95% CI)	*P* value
**Hospital-level effects**		
Hospital overall imaging rate	9.01 (1.5-54.24)	.02
Hospital percent Hispanic and non-Hispanic Black	1.00 (1.00-1.01)	.76
**Patient-level effects**		
Race and ethnicity		
Non-Hispanic Black	0.75 (0.75-0.76)	<.001
Non-Hispanic White	1 [Reference]	
Age, y		
<1	0.37 (0.36-0.37)	<.001
1-4	0.44 (0.44-0.44)	<.001
5-12	0.79 (0.79-0.80)	<.001
13-18	1 [Reference]	
Sex: female vs male	1.13 (1.12-1.15)	<.001
Insurance		
Public	1.05 (1.01-1.09)	.01
Private	1.3 (1.25-1.35)	<.001
Other	1 [Reference]	
Weekday vs weekend	1.05 (1.02-1.07)	<.001
ED arrival		
8:00 am to 3:59 pm	1.39 (1.34-1.45)	<.001
4:00 pm to 11:59 pm	1.37 (1.32-1.43)	<.001
12:00 am to 7:59 am	1 [Reference]	
Distance from hospital, miles		
>20	0.82 (0.8-0.85)	<.001
10-19	0.86 (0.84-0.89)	<.001
5-9	0.94 (0.91-0.97)	<.001
<5	1 [Reference]	
Year		
2016	0.92 (0.95-0.97)	<.001
2017	0.96 (0.05-0.97)	<.001
2018	0.96 (0.96-0.97)	<.001
2019	1 [Reference]	
Admission: no vs yes	0.42 (0.42-0.43)	<.001
ICU utilization: no vs yes	0.32 (0.31-0.33)	<.001
CCC: no vs yes	0.66 (0.66-0.67)	<.001
3 d return visit: no vs yes	0.02 (0.01-0.02)	<.001

## Discussion

Our multicenter cross-sectional study found that an increased proportion of Hispanic and non-Hispanic Black patients at US children’s hospitals was correlated with more pronounced racial differences in imaging rates. Most notably, as the percentage of children from minoritized groups cared for by a hospital increased, the degree of difference in imaging between non-Hispanic Black and non-Hispanic White children increased, with non-Hispanic White children more likely to receive imaging. Several of our findings suggest differential treatment of individuals by race within hospitals rather than differential patterns of presentation to hospitals: (1) the consistency of differences in imaging by race across hospitals, (2) increasing differences in hospitals with higher percentages of patients from minoritized groups and (3) the lack of association with hospital mean imaging rates.

Although it is possible that a portion of this variation may be due to differences in the case mix of disease severity or indications for imaging by hospital, all models adjusted for diagnosis-related group as well as other potential confounders. Importantly, these data do not provide an indication of whether this represents overtesting of 1 group or undertesting of another. However, prior work suggests a substantial role for overtesting of non-Hispanic White patients for head CT and for chest pain, ^2,20^ as well as differential patterns of overtreatment of non-Hispanic White patients with bronchiolitis^19^ or viral upper respiratory tract infections.^11^ Although overtesting carries risks including radiation exposure,^20^ increased length of stay,^21^ downstream effects of false positives, and costs to payers and patients,^22^ it is important to view this in the broader context in which children from minoritized groups consistently experience worse health outcomes compared with their White peers. Regardless of the directionality of the differences, the finding of differential imaging in all hospitals highlights the pervasive presence of structural and individual biases and inequities in care.

It remains concerning that increased differences in imaging by race and ethnicity are seen at hospitals that care for higher volumes of patients from minoritized groups, and additional work is needed to better understand the drivers of these differential patterns of imaging and develop interventions to improve the quality and equity of ED care around imaging decisions. Additional work is also needed to investigate patterns of imaging differences among other children from other racial and ethnic groups. To start with, hospitals should measure their own differences in imaging rates and increase awareness of existing areas of differential treatment as a starting point for improvement. In particular, the development of (and adherence to) evidence-based guidelines^23^ may help mitigate some biases in clinician evaluation and decision-making.^24^ However, more research is needed to determine the optimal implementation strategies for such guidelines and to demonstrate an impact on utilization and outcomes of care.

### Limitations

Limitations of this work include the absence of comprehensive clinical data regarding both indications for imaging, illness severity and whether a patient was referred in for imaging. However, the described differences persisted even after adjustment for relevant confounders. Additionally, our prior work demonstrated that racial disparities in imaging were, in fact, larger in nonhospitalized children,^9^ compared with those who were hospitalized from the ED, making it unlikely that higher imaging rates in non-Hispanic White children were due to higher severity of disease, although unmeasured case-mix differences cannot be fully excluded from these data. Furthermore, we do not have data on clinician characteristics, and are unable to determine variation at the level of the attending physician. Limitations of the sensitivity analysis include that we were unable to fit the model with random effects at the patient level using the full data set and so were limited to investigating imaging disparities at the first visit only. Additionally, these data are drawn from children’s hospitals and these patterns of care may not generalize to other emergency department settings. Together, however, the correlation data and the mixed-effects model demonstrate a persistent pattern of disparities in imaging across hospitals that does not appear to be associated with hospital level variation in imaging propensity and warrants further investigation.

## Conclusions

Overall, these data show differences in imaging rates by race and ethnicity across children’s hospitals, and suggest that hospitals with a higher percentage of pediatric patients from minoritized groups have larger differences in imaging between non-Hispanic Black and White patients. Additionally, these data do not support the hypothesis that racial and ethnic differences in imaging are associated with underlying variation in hospital propensity to image, emphasizing the need for additional work to develop interventions to improve the equity and appropriateness of imaging in pediatric emergency medicine.

## Notes

Supplement. **eTable 1.** Unadjusted Odds Ratios for Imaging by Race and Ethnicity for Each Hospital, Overall and for Specific Imaging Modalities **eTable 2.** Adjusted Odds Ratios for Imaging by Race and Ethnicity for Each Hospital, Overall and for Specific Imaging Modalities **eFigure.** Inclusion of Hospitals in the Cohort
