# Supplementary material for: Analysis of Racial and Ethnic Diversity of Population Served and Imaging Used in US Children’s Hospital Emergency Departments
Source: JAMA Netw Open. 2022 Jun 2;5(6):e2213951. doi: 10.1001/jamanetworkopen.2022.13951 (PMC9164005; doi:10.1001/jamanetworkopen.2022.13951)
Supplement: Supplement. — eTable 1. Unadjusted Odds Ratios for Imaging by Race and Ethnicity for Each Hospital, Overall and for Specific Imaging Modalities eTable 2. Adjusted Odds Ratios for Imaging by Race and Ethnicity for Each Hospital, Overall and for Specific Imaging Modalities eFigure. Inclusion of Hospitals in the Cohort [file jamanetwopen-e2213951-s001.pdf]

Supplemental Online Content

Samuels-Kalow ME, De Souza HG, Neuman MI, et al. Analysis of racial and ethnic diversity of population served and imaging used in US children's hospital emergency departments. *JAMA Netw Open*. 2022;5(6):e2213951. doi:10.1001/jamanetworkopen.2022.13951

eTable 1. Unadjusted Odds Ratios for Imaging by Race and Ethnicity for Each Hospital, Overall and for Specific Imaging Modalities
eTable 2. Adjusted Odds Ratios for Imaging by Race and Ethnicity for Each Hospital, Overall and for Specific Imaging Modalities
eFigure. Inclusion of Hospitals in the Cohort

This supplemental material has been provided by the authors to give readers additional information about their work.

© 2022 Samuels-Kalow ME et al. *JAMA Network Open.*

eTable 1. Unadjusted Odds Ratios for Imaging by Race and Ethnicity for Each Hospital, Overall and for Specific Imaging Modalities

Each row represents an individual hospital and shows the hospital demographic characteristics (population served) and the unadjusted odds ratios across all 3 racial/ethnic groups. Hospitals are ordered based on the percentage of non-White patients in their population served.

	Hospital Demographics (Population Served)		**Unadjusted OR for Imaging NH (Black as compared to White) and 95% CI**					**Unadjusted OR for Imaging (Hispanic as compared to NH Black) and 95% CI**					**Unadjusted OR for Imaging (Hispanic as compared to NH White) and 95% CI**				
**Hospital**	**% Non-White**	**% Black**	**Any imaging**	**X-ray**	**CT**	**US**	**MRI**	**Any imaging**	**X-ray**	**CT**	**US**	**MRI**	**Any imaging**	**X-ray**	**CT**	**US**	**MRI**
A	30.5	12.3	0.82 (0.80, 0.84)	0.94 (0.91, 0.96)	0.76 (0.72, 0.80)	0.59 (0.56, 0.63)	0.75 (0.65, 0.87)	1.02 (0.99, 1.05)	0.97 (0.94, 1.00)	0.87 (0.81, 0.94)	1.31 (1.23, 1.40)	0.84 (0.70, 1.01)	0.84 (0.82, 0.86)	0.91 (0.89, 0.93)	0.66 (0.63, 0.70)	0.78 (0.75, 0.81)	0.63 (0.55, 0.72)
B	32.5	14.4	0.74 (0.71, 0.77)	0.85 (0.82, 0.88)	0.74 (0.66, 0.83)	0.63 (0.59, 0.67)	0.61 (0.53, 0.70)	1.10 (1.04, 1.17)	1.02 (0.95, 1.09)	0.83 (0.69, 1.02)	1.33 (1.21, 1.46)	1.50 (1.23, 1.83)	0.82 (0.78, 0.86)	0.86 (0.82, 0.91)	0.62 (0.52, 0.73)	0.83 (0.77, 0.90)	0.92 (0.78, 1.08)
C	37.7	30.7	0.61 (0.60, 0.62)	0.75 (0.74, 0.77)	0.58 (0.56, 0.61)	0.43 (0.42, 0.45)	0.45 (0.42, 0.49)	1.17 (1.10, 1.24)	1.06 (1.00, 1.13)	1.01 (0.85, 1.19)	1.70 (1.53, 1.88)	1.58 (1.24, 2.01)	0.71 (0.67, 0.75)	0.80 (0.75, 0.85)	0.59 (0.50, 0.69)	0.74 (0.67, 0.81)	0.71 (0.56, 0.90)
D	38.0	9.3	0.81 (0.78, 0.84)	0.85 (0.82, 0.89)	0.62 (0.54, 0.70)	0.67 (0.60, 0.74)	0.61 (0.50, 0.76)	1.17 (1.12, 1.22)	1.12 (1.07, 1.17)	1.32 (1.14, 1.53)	1.63 (1.45, 1.83)	1.32 (1.04, 1.67)	0.95 (0.92, 0.97)	0.96 (0.93, 0.99)	0.81 (0.75, 0.89)	1.09 (1.02, 1.16)	0.81 (0.70, 0.93)
E	38.2	36.0	0.71 (0.70, 0.73)	0.85 (0.83, 0.87)	0.64 (0.61, 0.68)	0.48 (0.45, 0.50)	0.47 (0.43, 0.52)	1.07 (0.81, 1.42)	0.98 (0.72, 1.32)	1.12 (0.61, 2.05)	1.99 (1.18, 3.36)	2.46 (1.09, 5.56)	0.76 (0.58, 1.01)	0.83 (0.61, 1.12)	0.72 (0.39, 1.32)	0.95 (0.56, 1.60)	1.17 (0.52, 2.63)
F	41.1	3.2	0.71 (0.66, 0.76)	0.81 (0.76, 0.87)	0.68 (0.59, 0.79)	0.59 (0.51, 0.67)	0.69 (0.49, 0.98)	0.92 (0.86, 0.99)	0.86 (0.80, 0.93)	0.87 (0.75, 1.01)	1.22 (1.05, 1.41)	0.58 (0.40, 0.84)	0.65 (0.64, 0.67)	0.70 (0.68, 0.72)	0.59 (0.56, 0.63)	0.71 (0.68, 0.75)	0.40 (0.34, 0.47)
G	42.1	27.7	0.63 (0.62, 0.65)	0.70 (0.69, 0.71)	0.56 (0.54, 0.59)	0.43 (0.41, 0.45)	0.41 (0.34, 0.50)	1.05 (1.01, 1.09)	1.07 (1.03, 1.11)	0.83 (0.74, 0.93)	1.23 (1.10, 1.38)	0.92 (0.55, 1.53)	0.67 (0.64, 0.69)	0.75 (0.72, 0.77)	0.47 (0.42, 0.53)	0.53 (0.48, 0.59)	0.38 (0.24, 0.62)
H	42.8	28.1	0.59 (0.58, 0.60)	0.65 (0.65, 0.66)	0.60 (0.58, 0.63)	0.45 (0.44, 0.46)	0.39 (0.33, 0.46)	1.06 (1.03, 1.09)	0.99 (0.96, 1.02)	0.97 (0.89, 1.06)	1.52 (1.44, 1.60)	1.38 (1.03, 1.85)	0.62 (0.61, 0.64)	0.65 (0.63, 0.66)	0.59 (0.54, 0.64)	0.68 (0.65, 0.72)	0.54 (0.42, 0.70)
I	48.3	31.3	0.56 (0.55, 0.58)	0.67 (0.66, 0.69)	0.50 (0.47, 0.53)	0.45 (0.43, 0.47)	0.40 (0.35 0.46)	1.12 (1.08, 1.17)	1.04 (1.00, 1.09)	1.07 (0.98, 1.18)	1.63 (1.51, 1.75)	1.01 (0.81, 1.28)	0.64 (0.61, 0.66)	0.71 (0.68, 0.73)	0.54 (0.49, 0.59)	0.73 (0.68, 0.78)	0.41 (0.33, 0.50)
J	49.4	32.1	0.56 (0.55, 0.57)	0.63 (0.62, 0.64)	0.47 (0.45, 0.49)	0.45 (0.43, 0.48)	0.35 (0.31, 0.39)	1.05 (1.02, 1.08)	1.03 (1.01, 1.06)	1.01 (0.95, 1.08)	1.40 (1.30, 1.17)	1.02 (0.82, 1.26)	0.59 (0.57, 0.60)	0.65 (0.64, 0.67)	0.48 (0.45, 0.51)	0.64 (0.60, 0.68)	0.35 (0.29, 0.43)

© 2022 Samuels-Kalow ME et al. *JAMA Network Open.*

Hospital	% Non-White	% Black	Any imaging	X-ray	CT	US	MRI	Any imaging	X-ray	CT	US	MRI	Any imaging	X-ray	CT	US	MRI
K	50.2	32.3	0.59 (0.59, 0.60)	0.64 (0.63, 0.64)	0.56 (0.54, 0.59)	0.46 (0.45, 0.48)	0.43 (0.39, 0.48)	0.98 (0.95, 1.01)	0.93 (0.91, 0.96)	0.80 (0.71, 0.91)	1.47 (1.37, 1.57)	0.76 (0.54, 1.059)	0.58 (0.57, 0.60)	0.59 (0.58, 0.61)	0.45 (0.40, 0.51)	0.68 (0.64, 0.73)	0.33 (0.28, 0.45)
L	53.9	35.8	0.60 (0.59, 0.61)	0.71 (0.70, 0.72)	0.462 (0.44, 0.48)	0.41 (0.39, 0.43)	0.32 (0.28, 0.37)	1.10 (1.07, 1.13)	1.04 (1.01, 1.07)	1.18 (1.10, 1.27)	1.51 (1.41, 1.61)	1.34 (1.08, 1.66)	0.66 (0.64, 0.67)	0.74 (0.72, 0.76)	0.55 (0.51, 0.58)	0.62 (0.59, 0.66)	0.43 (0.36, 0.52)
M	55.9	8.4	0.67 (0.65, 0.68)	0.68 (0.66, 0.70)	0.76 (0.70, 0.82)	0.76 (0.71, 0.80)	0.64 (0.55, 0.76)	1.13 (1.10, 1.15)	1.10 (1.07, 1.13)	1.02 (0.94, 1.10)	1.22 (1.15, 1.29)	1.02 (0.86, 1.21)	0.75 (0.74, 0.76)	0.75 (0.74, 0.76)	0.77 (0.74, 0.81)	0.92 (0.89, 0.95)	0.66 (0.60, 0.72)
N	56.7	38.4	0.56 (0.55, 0.57)	0.68 (0.66, 0.70)	0.52 (0.49, 0.55)	0.43 (0.41, 0.45)	0.35 (0.32, 0.38)	1.26 (1.21, 1.31)	1.13 (1.08, 1.18)	1.35 (1.22, 1.49)	1.82 (1.70, 1.95)	1.80 (1.56, 2.07)	0.71 (0.68, 0.73)	0.77 (0.74, 0.80)	0.70 (0.64, 0.77)	0.78 (0.73, 0.83)	0.63 (0.55, 0.71)
O	58.5	51.2	0.65 (0.63, 0.66)	0.76 (0.74, 0.77)	0.46 (0.45, 0.48)	0.36 (0.34, 0.38)	0.42 (0.38, 0.46)	1.22 (1.18, 1.26)	1.14 (1.11, 1.19)	1.36 (1.27, 1.47)	1.96 (1.77, 2.17)	1.13 (0.9, 1.43)	0.78 (0.76, 0.81)	0.87 (0.84, 0.90)	0.63 (0.59, 0.68)	0.71 (0.65, 0.79)	0.47 (0.38, 0.59)
P	61.0	12.1	0.59 (0.57, 0.61)	0.72 (0.69, 0.75)	0.46 (0.41, 0.51)	0.53 (0.49, 0.56)	0.35 (0.28, 0.44)	1.18 (1.14, 1.23)	1.06 (1.02, 1.11)	1.28 (1.13, 1.45)	1.42 (1.32, 1.52)	1.69 (1.31, 2.17)	0.70 (0.68, 0.72)	0.76 (0.74, 0.79)	0.59 (0.54, 0.64)	0.75 (0.71, 0.78)	0.59 (0.51, 0.68)
Q	61.6	10.0	0.78 (0.76, 0.80)	0.91 (0.88, 0.94)	0.69 (0.64, 0.74)	0.59 (0.56, 0.63)	0.65 (0.58, 0.73)	1.01 (0.98, 1.03)	0.94 (0.91, 0.97)	0.95 (0.89, 1.02)	1.29 (1.22, 1.37)	0.70 (0.62, 0.79)	0.79 (0.77, 0.80)	0.86 (0.84, 0.88)	0.66 (0.63, 0.68)	0.76 (0.74, 0.79)	0.46 (0.43, 0.49)
R	63.7	17.2	0.51 (0.49, 0.52)	0.66 (0.64, 0.68)	0.33 (0.29, 0.37)	0.41 (0.38, 0.43)	0.28 (0.25, 0.319)	0.96 (0.93, 0.99)	0.91 (0.88, 0.94)	1.00 (0.88, 1.14)	1.19 (1.12, 1.26)	1.10 (0.96, 1.26)	0.49 (0.48, 0.5)	0.60 (0.59, 0.62)	0.33 (0.30, 0.36)	0.48 (0.46, 0.50)	0.31 (0.28, 0.34)
S	65.5	59.2	0.47 (0.46, 0.48)	0.59 (0.57, 0.60)	0.37 (0.35, 0.39)	0.27 (0.26, 0.29)	0.22 (0.20, 0.25)	1.29 (1.20, 1.37)	1.15 (1.07, 1.23)	1.60 (1.33, 1.92)	2.24 (1.95, 2.57)	2.05 (1.47, 2.86)	0.61 (0.57, 0.65)	0.68 (0.63, 0.73)	0.59 (0.49, 0.70)	0.61 (0.53, 0.70)	0.45 (0.32, 0.62)
T	67.4	34.8	0.53 (0.52, 0.54)	0.68 (0.67, 0.70)	0.34 (0.32, 0.36)	0.29 (0.28, 0.3)	0.23 (0.20, 0.26)	1.00 (0.97, 1.019)	0.94 (0.92, 0.97)	1.08 (1.00, 1.16)	1.45 (1.38, 1.53)	1.11 (0.92, 1.34)	0.53 (0.52, 0.54)	0.64 (0.63, 0.66)	0.36 (0.34, 0.39)	0.42 (0.4, 0.44)	0.26 (0.22, 0.30)
U	68.4	21.2	0.668 (0.657, 0.68)	0.819 (0.80, 0.83)	0.57 (0.54, 0.59)	0.44 (0.43, 0.46)	0.44 (0.40, 0.49)	1.12 (1.10, 1.14)	1.04 (1.03, 1.06)	1.05 (1.01, 1.10)	1.51 (1.46, 1.56)	1.12 (1.00, 1.25)	0.75 (0.74, 0.76)	0.86 (0.84, 0.87)	0.59 (0.57, 0.61)	0.67 (0.65, 0.68)	0.49 (0.45, 0.53)
V	70.0	26.9	0.53 (0.51, 0.55)	0.698 (0.67, 0.72)	0.46 (0.41, 0.51)	0.40 (0.37, 0.42)	0.33 (0.28, 0.38)	1.04 (1.01, 1.07)	0.96 (0.92, 0.99)	1.02 (0.91, 1.14)	1.32 (1.24, 1.40)	1.12 (0.96, 1.32)	0.55 (0.54, 0.57)	0.67 (0.65, 0.69)	0.47 (0.43, 0.51)	0.52 (0.50, 0.55)	0.37 (0.33, 0.42)
W	71.2	44.0	0.57 (0.568, 0.58)	0.68 (0.67, 0.69)	0.52 (0.51, 0.54)	0.44 (0.43, 0.45)	0.61 (0.56, 0.66)	0.97 (0.96, 0.99)	0.93 (0.91, 0.94)	0.95 (0.92, 0.99)	1.24 (1.21, 1.28)	0.54 (0.48, 0.61)	0.56 (0.55, 0.57)	0.63 (0.62, 0.64)	0.50 (0.48, 0.52)	0.55 (0.53, 0.57)	0.33 (0.29, 0.37)
X	72.4	43.2	0.498 (0.488, 0.509)	0.63 (0.62, 0.65)	0.51 (0.49, 0.54)	0.33 (0.32, 0.35)	0.31 (0.28, 0.35)	1.06 (1.03, 1.09)	0.96 (0.94, 0.99)	0.94 (0.88, 1.00)	1.59 (1.51, 1.68)	0.99 (0.85, 1.15)	0.53 (0.51, 0.54)	0.61 (0.59, 0.62)	0.48 (0.46, 0.51)	0.53 (0.50, 0.56)	0.31 (0.27, 0.35)
Y	72.4	17.3	0.56 (0.549, 0.58)	0.73 (0.71, 0.75)	0.45 (0.41, 0.49)	0.37 (0.35, 0.39)	0.30 (0.23, 0.39)	0.93 (0.91, 0.96)	0.88 (0.86, 0.91)	0.89 (0.81, 0.97)	1.237 (1.17, 1.31)	0.87 (0.65, 1.16)	0.53 (0.52, 0.54)	0.64 (0.63, 0.66)	0.40 (0.37, 0.42)	0.46 (0.44, 0.48)	0.26 (0.22, 0.32)

© 2022 Samuels-Kalow ME et al. *JAMA Network Open.*

Z	76.3	19.0	0.756 (0.74, 0.77)	0.91 (0.89, 0.93)	0.74 (0.71, 0.77)	0.61 (0.59, 0.63)	0.71 (0.65, 0.79)	1.04 (1.02, 1.06)	0.91 (0.89, 0.92)	0.91 (0.87, 0.94)	1.46 (1.42, 1.51)	0.76 (0.69, 0.84)	0.79 (0.78, 0.80)	0.82 (0.81, 0.84)	0.67 (0.65, 0.69)	0.89 (0.87, 0.91)	0.54 (0.50, 0.59)

Hospital	% Non-White	% Black	Any imaging	X-ray	CT	US	MRI	Any imaging	X-ray	CT	US	MRI	Any imaging	X-ray	CT	US	MRI
AA	77.1	56.4	0.45 (0.45, 0.46)	0.60 (0.59, 0.61)	0.37 (0.35, 0.39)	0.33 (0.32, 0.34)	0.21 (0.20, 0.23)	1.38 (1.34, 1.41)	1.25 (1.22, 1.28)	1.51 (1.40, 1.62)	1.71 (1.64, 1.79)	1.98 (1.75, 2.24)	0.62 (0.61, 0.64)	0.75 (0.73, 0.77)	0.56 (0.52, 0.60)	0.57 (0.55, 0.60)	0.42 (0.37, 0.47)
BB	78.8	15.0	0.65 (0.63, 0.67)	0.78 (0.76, 0.81)	0.57 (0.51, 0.63)	0.52 (0.49, 0.56)	0.43 (0.39, 0.48)	0.92 (0.89, 0.95)	0.86 (0.83, 0.89)	0.81 (0.73, 0.89)	1.18 (1.12, 1.25)	0.93 (0.85, 1.03)	0.60 (0.58, 0.61)	0.67 (0.66, 0.69)	0.46 (0.43, 0.49)	0.62 (0.60, 0.65)	0.41 (0.38, 0.43)
CC	79.0	4.9	0.80 (0.78, 0.83)	0.88 (0.85, 0.90)	0.63 (0.57, 0.69)	0.61 (0.56, 0.65)	0.57 (0.44, 0.74)	0.90 (0.87, 0.92)	0.85 (0.82, 0.87)	1.06 (0.97, 1.16)	1.33 (1.24, 1.42)	1.08 (0.84, 1.39)	0.72 (0.71, 0.73)	0.74 (0.73, 0.76)	0.67 (0.64, 0.70)	0.80 (0.78, 0.83)	0.62 (0.55, 0.69)
DD	79.2	1.6	0.94 (0.89, 1.00)	1.07 (1.00, 1.14)	0.72 (0.62, 0.84)	0.81 (0.74, 0.89)	0.73 (0.49, 1.09)	0.80 (0.75, 0.84)	0.77 (0.72, 0.82)	0.84 (0.73, 0.98)	0.95 (0.87, 1.04)	0.62 (0.42, 0.93)	0.75 (0.74, 0.76)	0.82 (0.80, 0.84)	0.61 (0.59, 0.64)	0.77 (0.75, 0.79)	0.45 (0.40, 0.51)
EE	80.3	23.0	0.62 (0.60, 0.65)	0.80 (0.76, 0.83)	0.59 (0.53, 0.67)	0.42 (0.38, 0.46)	0.47 (0.40, 0.54)	1.21 (1.17, 1.26)	1.10 (1.06, 1.147)	1.25 (1.13, 1.39)	1.73 (1.59, 1.87)	1.08 (0.95, 1.24)	0.758 (0.73, 0.79)	0.88 (0.84, 0.91)	0.74 (0.68, 0.82)	0.72 (0.68, 0.77)	0.51 (0.45, 0.57)
FF	81.2	21.4	0.61 (0.60, 0.62)	0.72 (0.70, 0.73)	0.51 (0.49, 0.53)	0.48 (0.46, 0.49)	0.52 (0.46, 0.58)	1.08 (1.07, 1.10)	1.03 (1.01, 1.04)	0.99 (0.95, 1.02)	1.46 (1.42, 1.50)	0.67 (0.60, 0.76)	0.66 (0.65, 0.67)	0.74 (0.73, 0.75)	0.50 (0.49, 0.52)	0.70 (0.68, 0.71)	0.35 (0.32, 0.39)
GG	82.2	73.0	0.40 (0.39, 0.41)	0.50 (0.49, 0.51)	0.37 (0.36, 0.38)	0.29 (0.28, 0.30)	0.22 (0.20, 0.24)	1.26 (1.06, 1.50)	1.22 (1.01, 1.47)	0.89 (0.57, 1.38)	2.16 (1.51, 3.08)	0.47 (0.06, 3.35)	0.51 (0.43, 0.60)	0.61 (0.51, 0.74)	0.33 (0.21, 0.51)	0.63 (0.44, 0.9)	0.10 (0.01, 0.74)
HH	84.0	4.0	0.73 (0.70, 0.76)	0.80 (0.77, 0.84)	0.69 (0.62, 0.77)	0.64 (0.60, 0.69)	0.61 (0.50, 0.76)	0.94 (0.91, 0.97)	0.88 (0.85, 0.91)	0.90 (0.81, 0.99)	1.21 (1.13, 1.29)	0.81 (0.66, 1.00)	0.69 (0.68, 0.70)	0.71 (0.70, 0.72)	0.62 (0.59, 0.65)	0.78 (0.75, 0.80)	0.50 (0.46, 0.55)
II	84.4	28.6	0.57 (0.54, 0.6)	0.72 (0.69, 0.76)	0.52 (0.48, 0.57)	0.53 (0.48, 0.58)	0.39 (0.35, 0.45)	1.22 (1.17, 1.27)	1.08 (1.04, 1.12)	1.10 (1.01, 1.19)	1.79 (1.66, 1.936)	1.78 (1.59, 2.00)	0.69 (0.66, 0.73)	0.78 (0.75, 0.82)	0.57 (0.53, 0.62)	0.95 (0.88, 1.02)	0.70 (0.63, 0.78)
JJ	87.5	53.2	0.68 (0.66, 0.70)	0.83 (0.80, 0.86)	0.59 (0.55, 0.63)	0.60 (0.57, 0.63)	0.36 (0.32, 0.40)	0.91 (0.86, 0.96)	0.83 (0.77, 0.88)	0.50 (0.40, 0.61)	1.31 (1.20, 1.44)	0.29 (0.18, 0.49)	0.62 (0.58, 0.66)	0.68 (0.64, 0.73)	0.29 (0.24, 0.36)	0.79 (0.72, 0.87)	0.10 (0.06, 0.17)
KK	90.8	22.1	0.47 (0.45, 0.49)	0.58 (0.56, 0.61)	0.36 (0.32, 0.40)	0.35 (0.32, 0.38)	0.29 (0.24, 0.35)	1.12 (1.09, 1.16)	1.01 (0.98, 1.05)	1.18 (1.07, 1.30)	1.79 (1.67, 1.92)	1.13 (0.97, 1.33)	0.53 (0.51, 0.55)	0.59 (0.57, 0.61)	0.42 (0.38, 0.46)	0.62 (0.58, 0.66)	0.33 (0.29, 0.38)
LL	95.5	6.1	0.55 (0.52, 0.58)	0.63 (0.60, 0.67)	0.51 (0.45, 0.58)	0.49 (0.45, 0.54)	0.35 (0.29, 0.42)	0.96 (0.93, 0.99)	0.91 (0.88, 0.95)	0.88 (0.80, 0.97)	1.24 (1.15, 1.34)	0.74 (0.63, 0.88)	0.53 (0.51, 0.55)	0.58 (0.56, 0.60)	0.45 (0.41, 0.49)	0.61 (0.57, 0.65)	0.26 (0.23, 0.29)

© 2022 Samuels-Kalow ME et al. *JAMA Network Open.*

eTable 2. Adjusted Odds Ratios for Imaging by Race and Ethnicity for Each Hospital, Overall and for Specific Imaging Modalities

Each row represents an individual hospital and shows the hospital demographic characteristics (population served) and the adjusted odds ratios across all 3 racial/ethnic groups.

	Hospital demographics		Adjusted OR for Imaging (Black compared to White) and 95% CI					Adjusted OR for Imaging (Hispanic compared to Black) and 95% CI					Adjusted OR for Imaging (Hispanic compared to White) and 95% CI				
Hospital	% Non-White	% Black	Any imaging	X-ray	CT	US	MRI	Any imaging	X-ray	CT	US	MRI	Any imaging	X-ray	CT	US	MRI
A	30.5	12.3	0.89 (0.87, 0.92)	0.98 (0.95, 1.01)	0.90 (0.85, 0.95)	0.69 (0.65, 0.73)	1.04 (0.89, 1.21)	1.06 (1.03, 1.10)	1.00 (0.96, 1.03)	0.92 (0.85, 1.00)	1.39 (1.30, 1.49)	0.9 (0.74, 1.10)	0.94 (0.92, 0.97)	0.97 (0.94, 0.99)	0.84 (0.79, 0.89)	0.95 (0.91, 1.00)	0.92 (0.80, 1.07)
B	32.5	14.4	0.85 (0.82, 0.89)	0.94 (0.90, 0.99)	0.91 (0.81, 1.02)	0.75 (0.70, 0.80)	0.79 (0.68, 0.91)	1.12 (1.05, 1.19)	1.03 (0.96, 1.1)	0.83 (0.68, 1.02)	1.34 (1.21, 1.48)	1.53 (1.25, 1.88)	0.94 (0.89, 1.00)	0.95 (0.90, 1.01)	0.75 (0.63, 0.89)	0.99 (0.91, 1.07)	1.2 (1.02, 1.42)
C	37.7	30.7	0.77 (0.76, 0.79)	0.87 (0.85, 0.89)	0.82 (0.78, 0.86)	0.60 (0.58, 0.63)	0.74 (0.68, 0.81)	1.09 (1.02, 1.16)	1.01 (0.94, 1.08)	0.90 (0.75, 1.07)	1.48 (1.33, 1.65)	1.17 (0.91, 1.50)	0.85 (0.80, 0.90)	0.89 (0.83, 0.95)	0.75 (0.64, 0.89)	0.91 (0.83, 1.01)	0.94 (0.74, 1.20)
D	38.0	9.3	0.85 (0.81, 0.89)	0.9 (0.87, 0.95)	0.66 (0.57, 0.76)	0.69 (0.61, 0.77)	0.78 (0.62, 0.98)	1.21 (1.15, 1.27)	1.15 (1.09, 1.2)	1.33 (1.13, 1.56)	1.62 (1.43, 1.83)	1.28 (0.99, 1.65)	1.02 (0.99, 1.06)	1.03 (0.99, 1.06)	0.91 (0.82, 1.00)	1.14 (1.06, 1.23)	0.96 (0.82, 1.13)
E	38.2	36.0	0.88 (0.86, 0.90)	0.97 (0.95, 1.00)	0.88 (0.83, 0.92)	0.63 (0.59, 0.66)	0.85 (0.77, 0.94)	1.08 (0.80, 1.45)	1.00 (0.74, 1.37)	1.16 (0.62, 2.15)	1.90 (1.11, 3.24)	2.75 (1.18, 6.42)	0.94 (0.71, 1.26)	0.99 (0.73, 1.34)	0.99 (0.54, 1.82)	1.13 (0.66, 1.91)	2.1 (0.92, 4.82)
F	41.1	3.2	0.84 (0.79, 0.90)	0.92 (0.85, 0.99)	0.83 (0.72, 0.97)	0.74 (0.64, 0.85)	1.04 (0.73, 1.49)	1.02 (0.95, 1.10)	0.95 (0.87, 1.02)	1.00 (0.85, 1.17)	1.33 (1.14, 1.55)	0.74 (0.51, 1.08)	0.85 (0.83, 0.88)	0.86 (0.83, 0.89)	0.82 (0.77, 0.87)	0.95 (0.90, 1.01)	0.73 (0.62, 0.87)
G	42.1	27.7	0.80 (0.79, 0.82)	0.85 (0.84, 0.87)	0.73 (0.69, 0.76)	0.57 (0.54, 0.60)	0.67 (0.54, 0.84)	1.00 (0.96, 1.04)	1.00 (0.96, 1.04)	0.90 (0.80, 1.01)	1.43 (1.27, 1.62)	1.10 (0.64, 1.87)	0.79 (0.76, 0.82)	0.84 (0.81, 0.87)	0.63 (0.56, 0.70)	0.74 (0.67, 0.83)	0.67 (0.41, 1.09)
H	42.8	28.1	0.75 (0.74, 0.76)	0.81 (0.80, 0.83)	0.84 (0.80, 0.89)	0.62 (0.59, 0.64)	0.66 (0.54, 0.80)	1.11 (1.07, 1.14)	1.03 (1.00, 1.06)	0.98 (0.89, 1.09)	1.46 (1.37, 1.56)	1.45 (1.04, 2.03)	0.82 (0.80, 0.85)	0.83 (0.81, 0.85)	0.82 (0.75, 0.90)	0.91 (0.87, 0.96)	0.96 (0.73, 1.26)
I	48.3	31.3	0.73 (0.71, 0.75)	0.80 (0.77, 0.82)	0.75 (0.70, 0.79)	0.65 (0.62, 0.69)	0.75 (0.65, 0.87)	1.17 (1.11, 1.23)	1.08 (1.02, 1.13)	1.04 (0.93, 1.16)	1.53 (1.40, 1.66)	1.16 (0.89, 1.52)	0.83 (0.79, 0.87)	0.83 (0.79, 0.87)	0.78 (0.71, 0.86)	1.01 (0.94, 1.09)	0.71 (0.56, 0.89)
J	49.4	32.1	0.72 (0.70, 0.73)	0.78 (0.76, 0.79)	0.61 (0.59, 0.64)	0.63 (0.59, 0.66)	0.67 (0.59, 0.77)	1.07 (1.04, 1.11)	1.06 (1.02, 1.09)	0.99 (0.92, 1.06)	1.54 (1.42, 1.67)	0.99 (0.79, 1.26)	0.75 (0.73, 0.77)	0.79 (0.77, 0.82)	0.63 (0.59, 0.67)	0.89 (0.83, 0.96)	0.7 (0.57, 0.86)
K	50.2	32.3	0.76 (0.75, 0.77)	0.80 (0.79, 0.81)	0.78 (0.74, 0.83)	0.61 (0.59, 0.64)	0.90 (0.78, 1.03)	1 (0.97, 1.03)	0.95 (0.92, 0.98)	0.91 (0.80, 1.04)	1.59 (1.48, 1.70)	0.95 (0.68, 1.34)	0.74 (0.71, 0.76)	0.74 (0.72, 0.77)	0.68 (0.60, 0.77)	0.91 (0.84, 0.97)	0.81 (0.58, 1.13)
L	53.9	35.8	0.75 (0.73, 0.77)	0.85 (0.83, 0.87)	0.62 (0.59, 0.66)	0.60 (0.57, 0.63)	0.69 (0.59, 0.81)	1.12 (1.08, 1.15)	1.07 (1.03, 1.10)	1.16 (1.07, 1.25)	1.44 (1.34, 1.55)	1.33 (1.05, 1.67)	0.82 (0.79, 0.84)	0.87 (0.84, 0.89)	0.75 (0.70, 0.81)	0.83 (0.78, 0.89)	0.86 (0.71, 1.05)
M	55.9	8.4	0.79 (0.77, 0.81)	0.82 (0.80, 0.85)	0.84 (0.77, 0.93)	0.75 (0.70, 0.81)	0.92 (0.75, 1.11)	1.14 (1.12, 1.18)	1.10 (1.07, 1.13)	1.09 (1.00, 1.19)	1.34 (1.25, 1.43)	1.13 (0.95, 1.35)	0.87 (0.86, 0.89)	0.87 (0.85, 0.88)	0.92 (0.87, 0.97)	1.03 (0.99, 1.07)	0.95 (0.85, 1.06)

© 2022 Samuels-Kalow ME et al. *JAMA Network Open.*

N	56.7	38.4	0.71 (0.69, 0.73)	0.80 (0.78, 0.83)	0.73 (0.68, 0.78)	0.61 (0.58, 0.64)	0.66 (0.59, 0.73)	1.11 (1.05, 1.16)	1.02 (0.97, 1.07)	1.13 (1.00, 1.27)	1.48 (1.36, 1.62)	1.09 (0.91, 1.29)	0.79 (0.76, 0.83)	0.84 (0.80, 0.88)	0.80 (0.72, 0.88)	0.92 (0.86, 0.99)	0.70 (0.61, 0.81)

**Hospital**	**% Non-White**	**% Black**	**Any imaging**	**X-ray**	**CT**	**US**	**MRI**	**Any imaging**	**X-ray**	**CT**	**US**	**MRI**	**Any imaging**	**X-ray**	**CT**	**US**	**MRI**
O	58.5	51.2	0.81 (0.80, 0.83)	0.91 (0.89, 0.94)	0.63 (0.61, 0.66)	0.58 (0.55, 0.62)	0.83 (0.74, 0.94)	1.22 (1.18, 1.26)	1.15 (1.10, 1.19)	1.29 (1.19, 1.40)	1.83 (1.64, 2.04)	0.96 (0.76, 1.23)	0.95 (0.92, 0.99)	1.00 (0.96, 1.04)	0.82 (0.76, 0.89)	1.00 (0.90, 1.12)	0.80 (0.63, 1.02)
P	61.0	12.1	0.72 (0.69, 0.75)	0.81 (0.77, 0.85)	0.62 (0.55, 0.70)	0.68 (0.63, 0.73)	0.65 (0.51, 0.83)	1.12 (1.07, 1.17)	1.02 (0.97, 1.07)	1.18 (1.04, 1.34)	1.29 (1.20, 1.39)	1.34 (1.03, 1.74)	0.83 (0.81, 0.86)	0.86 (0.83, 0.90)	0.75 (0.69, 0.82)	0.89 (0.85, 0.94)	0.89 (0.76, 1.05)
Q	61.6	10.0	0.91 (0.88, 0.95)	1.02 (0.98, 1.06)	0.87 (0.80, 0.94)	0.73 (0.68, 0.78)	0.88 (0.77, 1.02)	1.05 (1.01, 1.08)	0.97 (0.93, 1.00)	1.05 (0.97, 1.14)	1.38 (1.29, 1.48)	0.93 (0.79, 1.08)	0.96 (0.94, 0.98)	0.99 (0.96, 1.01)	0.90 (0.86, 0.95)	0.99 (0.95, 1.03)	0.79 (0.71, 0.87)
R	63.7	17.2	0.69 (0.66, 0.72)	0.80 (0.76, 0.83)	0.59 (0.51, 0.67)	0.62 (0.58, 0.67)	0.54 (0.46, 0.62)	1.07 (1.03, 1.11)	0.99 (0.96, 1.03)	1.14 (0.98, 1.32)	1.32 (1.23, 1.42)	1.28 (1.10, 1.50)	0.74 (0.71, 0.76)	0.79 (0.76, 0.82)	0.66 (0.58, 0.74)	0.82 (0.77, 0.87)	0.65 (0.57, 0.73)
S	65.5	59.2	0.71 (0.69, 0.74)	0.82 (0.79, 0.85)	0.72 (0.66, 0.79)	0.48 (0.45, 0.51)	0.63 (0.54, 0.74)	1.20 (1.12, 1.29)	1.09 (1.01, 1.18)	1.28 (1.05, 1.56)	1.81 (1.56, 2.11)	1.26 (0.88, 1.80)	0.86 (0.80, 0.93)	0.89 (0.83, 0.97)	0.97 (0.80, 1.17)	0.87 (0.75, 1.00)	0.85 (0.61, 1.20)
T	67.4	34.8	0.73 (0.71, 0.75)	0.83 (0.81, 0.86)	0.55 (0.51, 0.59)	0.49 (0.47, 0.52)	0.57 (0.49, 0.67)	1.00 (0.98, 1.03)	0.95 (0.93, 0.97)	1.08 (1.00, 1.17)	1.49 (1.41, 1.58)	1.23 (1.01, 1.49)	0.74 (0.72, 0.76)	0.80 (0.78, 0.83)	0.57 (0.53, 0.62)	0.71 (0.68, 0.76)	0.67 (0.56, 0.81)
U	68.4	21.2	0.82 (0.8, 0.84)	0.93 (0.91, 0.95)	0.73 (0.69, 0.76)	0.60 (0.58, 0.63)	0.70 (0.62, 0.79)	1.16 (1.14, 1.18)	1.07 (1.05, 1.08)	1.10 (1.05, 1.15)	1.58 (1.53, 1.64)	1.27 (1.13, 1.42)	0.93 (0.92, 0.95)	0.98 (0.96, 0.99)	0.81 (0.78, 0.84)	0.92 (0.90, 0.95)	0.88 (0.80, 0.96)
V	70.0	26.9	0.71 (0.67, 0.74)	0.82 (0.78, 0.86)	0.65 (0.57, 0.75)	0.61 (0.57, 0.66)	0.59 (0.49, 0.72)	1.09 (1.05, 1.13)	0.99 (0.95, 1.03)	1.06 (0.94, 1.2)	1.39 (1.30, 1.49)	1.15 (0.97, 1.36)	0.78 (0.75, 0.82)	0.81 (0.77, 0.85)	0.75 (0.67, 0.85)	0.88 (0.82, 0.94)	0.79 (0.67, 0.92)
W	71.2	44.0	0.73 (0.72, 0.74)	0.82 (0.80, 0.83)	0.69 (0.67, 0.72)	0.63 (0.61, 0.65)	0.94 (0.85, 1.02)	1.10 (1.08, 1.12)	1.03 (1.01, 1.04)	1.09 (1.05, 1.14)	1.42 (1.37, 1.46)	0.70 (0.62, 0.79)	0.77 (0.76, 0.79)	0.79 (0.77, 0.80)	0.76 (0.72, 0.79)	0.88 (0.84, 0.91)	0.65 (0.57, 0.75)
X	72.4	43.2	0.70 (0.68, 0.73)	0.81 (0.78, 0.83)	0.79 (0.74, 0.85)	0.54 (0.50, 0.57)	0.67 (0.58, 0.78)	1.11 (1.08, 1.14)	1.01 (0.98, 1.04)	0.96 (0.90, 1.03)	1.60 (1.50, 1.69)	1.10 (0.93, 1.30)	0.76 (0.74, 0.79)	0.79 (0.76, 0.82)	0.77 (0.71, 0.83)	0.81 (0.76, 0.86)	0.72 (0.61, 0.85)
Y	72.4	17.3	0.76 (0.73, 0.79)	0.89 (0.86, 0.93)	0.68 (0.61, 0.76)	0.56 (0.53, 0.60)	0.66 (0.48, 0.91)	1.00 (0.97, 1.03)	0.94 (0.91, 0.96)	1.02 (0.93, 1.13)	1.34 (1.26, 1.42)	1.04 (0.77, 1.41)	0.81 (0.78, 0.83)	0.86 (0.84, 0.89)	0.72 (0.66, 0.79)	0.81 (0.77, 0.86)	0.69 (0.54, 0.87)
Z	76.3	19.0	0.82 (0.80, 0.84)	0.96 (0.93, 0.98)	0.81 (0.77, 0.85)	0.67 (0.65, 0.69)	0.88 (0.79, 0.98)	1.15 (1.13, 1.17)	0.98 (0.96, 1.00)	1.00 (0.96, 1.04)	1.60 (1.55, 1.65)	0.91 (0.82, 1.00)	0.91 (0.90, 0.93)	0.91 (0.89, 0.93)	0.81 (0.78, 0.85)	1.02 (1.00, 1.05)	0.78 (0.71, 0.86)
AA	77.1	56.4	0.64 (0.62, 0.65)	0.74 (0.72, 0.76)	0.69 (0.64, 0.74)	0.57 (0.55, 0.60)	0.57 (0.51, 0.63)	1.23 (1.20, 1.26)	1.13 (1.10, 1.17)	1.07 (0.98, 1.16)	1.42 (1.35, 1.48)	1.29 (1.13, 1.49)	0.77 (0.74, 0.79)	0.81 (0.78, 0.84)	0.75 (0.69, 0.81)	0.79 (0.75, 0.83)	0.75 (0.66, 0.85)
BB	78.8	15.0	0.74 (0.71, 0.78)	0.88 (0.84, 0.92)	0.75 (0.66, 0.85)	0.59 (0.55, 0.64)	0.53 (0.47, 0.60)	1.12 (1.08, 1.16)	1.01 (0.98, 1.05)	1.07 (0.96, 1.19)	1.45 (1.36, 1.54)	1.38 (1.25, 1.53)	0.82 (0.79, 0.85)	0.87 (0.83, 0.90)	0.78 (0.70, 0.87)	0.87 (0.82, 0.92)	0.74 (0.67, 0.81)

© 2022 Samuels-Kalow ME et al. *JAMA Network Open.*

Hospital	% Non-White	% Black	Any imaging	X-ray	CT	US	MRI	Any imaging	X-ray	CT	US	MRI	Any imaging	X-ray	CT	US	MRI
CC	79.0	4.9	0.83 (0.8, 0.86)	0.87 (0.84, 0.91)	0.77 (0.69, 0.85)	0.73 (0.68, 0.79)	0.77 (0.58, 1.02)	1.06 (1.03, 1.09)	1.00 (0.97, 1.04)	1.17 (1.06, 1.29)	1.44 (1.34, 1.55)	1.22 (0.94, 1.58)	0.83 (0.82, 0.85)	0.83 (0.82, 0.85)	0.86 (0.82, 0.90)	1.01 (0.98, 1.05)	0.92 (0.81, 1.05)
DD	79.2	1.6	0.91 (0.85, 0.98)	1.03 (0.96, 1.10)	0.67 (0.57, 0.79)	0.81 (0.74, 0.90)	0.61 (0.40, 0.93)	1.03 (0.96, 1.10)	0.92 (0.86, 0.99)	1.37 (1.16, 1.61)	1.27 (1.14, 1.41)	1.51 (0.99, 2.31)	0.93 (0.91, 0.95)	0.94 (0.92, 0.96)	0.85 (0.81, 0.90)	1.00 (0.97, 1.03)	0.90 (0.78, 1.03)
EE	80.3	23.0	0.68 (0.65, 0.72)	0.85 (0.80, 0.90)	0.70 (0.61, 0.80)	0.49 (0.45, 0.55)	0.53 (0.45, 0.62)	1.18 (1.09, 1.27)	0.98 (0.90, 1.07)	1.19 (0.99, 1.44)	1.88 (1.65, 2.15)	1.45 (1.16, 1.81)	0.90 (0.83, 0.97)	0.92 (0.85, 1.00)	0.90 (0.75, 1.08)	1.06 (0.94, 1.20)	0.84 (0.68, 1.03)
FF	81.2	21.4	0.74 (0.73, 0.76)	0.83 (0.82, 0.85)	0.69 (0.66, 0.73)	0.62 (0.60, 0.64)	0.89 (0.78, 1.02)	1.22 (1.20, 1.23)	1.13 (1.11, 1.15)	1.17 (1.12, 1.22)	1.62 (1.58, 1.67)	0.95 (0.84, 1.07)	0.88 (0.87, 0.90)	0.92 (0.90, 0.93)	0.81 (0.78, 0.85)	0.97 (0.94, 1.00)	0.84 (0.74, 0.95)
GG	82.2	73.0	0.64 (0.62, 0.66)	0.73 (0.71, 0.75)	0.72 (0.68, 0.75)	0.63 (0.60, 0.67)	0.83 (0.73, 0.94)	1.28 (1.06, 1.55)	1.27 (1.05, 1.55)	0.75 (0.48, 1.19)	1.71 (1.17, 2.50)	0.34 (0.05, 2.45)	0.80 (0.67, 0.97)	0.85 (0.70, 1.03)	0.57 (0.36, 0.89)	0.97 (0.67, 1.41)	0.31 (0.04, 2.25)
HH	84.0	4.0	0.8 (0.76, 0.84)	0.87 (0.83, 0.92)	0.76 (0.67, 0.87)	0.72 (0.65, 0.79)	0.78 (0.60, 1.01)	1.00 (0.96, 1.05)	0.92 (0.88, 0.96)	1.03 (0.91, 1.17)	1.32 (1.21, 1.44)	0.93 (0.73, 1.20)	0.79 (0.77, 0.81)	0.80 (0.78, 0.82)	0.76 (0.72, 0.81)	0.94 (0.90, 0.98)	0.71 (0.62, 0.80)
II	84.4	28.6	0.83 (0.78, 0.89)	0.96 (0.90, 1.03)	0.84 (0.75, 0.95)	0.68 (0.60, 0.76)	0.71 (0.60, 0.83)	1.19 (1.14, 1.24)	1.05 (1.01, 1.10)	1.03 (0.94, 1.13)	1.73 (1.59, 1.88)	1.73 (1.53, 1.96)	0.96 (0.90, 1.01)	0.96 (0.91, 1.02)	0.99 (0.89, 1.10)	1.10 (1.00, 1.22)	1.05 (0.92, 1.20)
JJ	87.5	53.2	0.81 (0.78, 0.84)	0.93 (0.89, 0.97)	0.77 (0.71, 0.84)	0.78 (0.74, 0.83)	0.58 (0.51, 0.66)	1.08 (1.01, 1.14)	0.93 (0.87, 1.00)	0.71 (0.57, 0.87)	1.76 (1.60, 1.93)	0.71 (0.42, 1.19)	0.72 (0.66, 0.78)	0.70 (0.64, 0.78)	0.41 (0.32, 0.52)	1.11 (0.97, 1.27)	0.28 (0.16, 0.49)
KK	90.8	22.1	0.63 (0.60, 0.67)	0.76 (0.72, 0.80)	0.69 (0.59, 0.79)	0.51 (0.46, 0.57)	0.54 (0.43, 0.66)	1.18 (1.14, 1.22)	1.06 (1.02, 1.10)	1.18 (1.07, 1.31)	1.83 (1.70, 1.97)	1.18 (1.00, 1.40)	0.78 (0.75, 0.83)	0.83 (0.79, 0.88)	0.84 (0.74, 0.95)	0.95 (0.87, 1.03)	0.69 (0.57, 0.82)
LL	95.5	6.1	0.72 (0.68, 0.77)	0.81 (0.76, 0.87)	0.82 (0.70, 0.95)	0.68 (0.60, 0.77)	0.59 (0.46, 0.74)	1.08 (1.04, 1.12)	1.00 (0.96, 1.04)	1.02 (0.92, 1.13)	1.44 (1.33, 1.56)	1.08 (0.91, 1.28)	0.82 (0.78, 0.86)	0.85 (0.81, 0.89)	0.85 (0.77, 0.95)	1.04 (0.96, 1.13)	0.78 (0.67, 0.91)

*adjusted for age, sex, weekend presentation, hour of presentation, insurance, hospital admission, intensive care unit admission, hospital site, complex chronic conditions, All Patient Refined–Diagnosis Related Group category, year, distance from hospital, and 3-day revisit

© 2022 Samuels-Kalow ME et al. *JAMA Network Open.*

eFigure. Inclusion of Hospitals in the Cohort

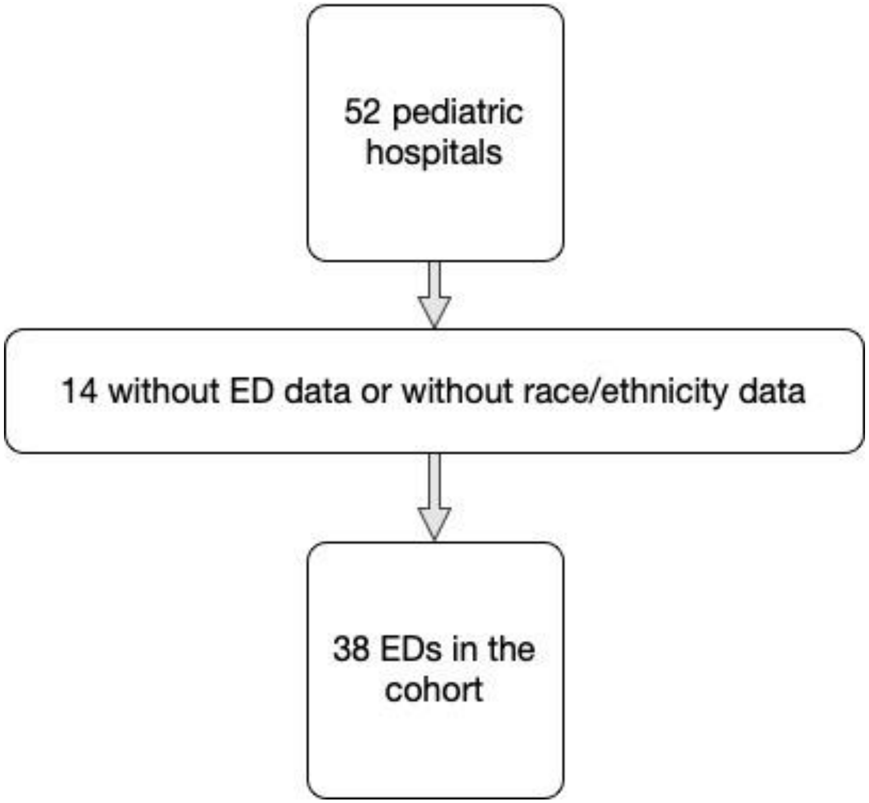

© 2022 Samuels-Kalow ME et al. *JAMA Network Open.*
